# Maize microRNA166 Inactivation Confers Plant Development and Abiotic Stress Resistance

**DOI:** 10.3390/ijms21249506

**Published:** 2020-12-14

**Authors:** Na Li, Tianxiao Yang, Zhanyong Guo, Qiusheng Wang, Mao Chai, Mingbo Wu, Xiaoqi Li, Weiya Li, Guangxian Li, Jihua Tang, Guiliang Tang, Zhanhui Zhang

**Affiliations:** 1 National Key Laboratory of Wheat and Maize Crop Science, Collaborative Innovation Center of Henan Grain Crops, College of Agronomy, Henan Agricultural University, Zhengzhou 450002, China; jiangxiaoyu0604@163.com (N.L.); tianxiao.yang@ufl.edu (T.Y.); gzy@henau.edu.cn (Z.G.); qxyler@outlook.com (Q.W.); chaimol@163.com (M.C.); wumingbo168@163.com (M.W.); xiaoqiLi1998@163.com (X.L.); liviya@126.com (W.L.); liguangxian27@163.com (G.L.); tangjihua@henau.edu.cn (J.T.); 2 Plant Molecular and Cellular Biology Program, University of Florida, Gainesville, FL 32611, USA; 3 Department of Biological Sciences and Biotechnology Research Center, Michigan Technological University, Houghton, MI 49931, USA

**Keywords:** maize, microRNA166 (miR166), short tandem target mimics (STTM), plant development, abiotic stress resistance

## Abstract

MicroRNAs are important regulators in plant developmental processes and stress responses. In this study, we generated a series of maize STTM166 transgenic plants. Knock-down of miR166 resulted in various morphological changes, including rolled leaves, enhanced abiotic stress resistance, inferior yield-related traits, vascular pattern and epidermis structures, tassel architecture, as well as abscisic acid (ABA) level elevation and indole acetic acid (IAA) level reduction in maize. To profile miR166 regulated genes, we performed RNA-seq and qRT-PCR analysis. A total of 178 differentially expressed genes (DEGs) were identified, including 118 up-regulated and 60 down-regulated genes. These DEGs were strongly enriched in cell and intercellular components, cell membrane system components, oxidoreductase activity, single organism metabolic process, carbohydrate metabolic process, and oxidation reduction process. These results indicated that miR166 plays important roles in auxin and ABA interaction in monocots, yet the specific mechanism may differ from dicots. The enhanced abiotic stress resistance is partly caused via rolling leaves, high ABA content, modulated vascular structure, and the potential changes of cell membrane structure. The inferior yield-related traits and late flowering are partly controlled by the decreased IAA content, the interplay of miR166 with other miRNAs and AGOs. Taken together, the present study uncovered novel functions of miR166 in maize, and provide insights on applying short tandem target mimics (STTM) technology in plant breeding.

## 1. Introduction

MicroRNAs (miRNA) are a class of endogenous small, single-stranded non-coding RNA molecules. In plants, the transcripts of *MIRNA* gene are firstly cleaved into pri-miRNAs by Dicer-like 1 (DCL1) protein, then are processed into 20–24 nt mature miRNAs. Further, mature miRNAs are recruited by Argonaute1 (AGO1) protein, and incorporated into RNA-induced silencing complexes (RISCs) [1]. The miRNA-RISCs serve to negatively regulate target gene expression via mRNA cleavage and degradation or translation inhibition [2,3,4]. miRNAs are essential to realize their functions through negatively regulating F-box proteins and transcription factors at post-transcriptional level [5]. It has been proven conserved miRNAs to modulate important plant biological processes, including development, immune responses, nutrient homeostasis, and hormone responses [6,7]. In particular, multiple agronomic traits in crops are controlled by different miRNA families [8,9].

miR165/166 family is a well-conserved and -characterized as a relatively abundant class of miRNAs in all land plants [10,11]. miR165 and miR166 have almost identical base sequences except for C-U at 17th base, which have been investigated extensively because of their unique mechanism of action [10]. In contrast to *MIR166* genes which are widely found in land plants, *MIR165* genes only have been found in the Brassicaceae family [12,13,14]. In *Arabidopsis*, miR165/166 family comprises of two miR165 (miR165a and miR165b) and seven miR166 (miR166a-miR166g) members, and has five target genes that encode HD-ZIP III transcription factors, such as PHABULOSA (PHB), PHAVOLUTA (PHV), REVOLUTA (REV), ATHB-8, and ATHB-15 [15,16,17]. Many studies have proposed that *Arabidopsis* miR165/166 plays critical roles in multiple developmental processes, such as shoot apical and lateral meristem initiation, leaf and shoot polarity formation, ovule morphogenesis, floral development, anthocyanin synthesis, and vascular patterning of shoot and root [11,14,18,19,20,21,22,23,24,25,26,27]. Moreover, the down-regulated of miR165/166 causes the up-regulated of its target gene, *PHB*, which in turn enhancing plant abiotic stresses resistance via abscisic acid (ABA) homeostasis regulation [28]. In the regulatory networks of different developmental processes, miR165/166 often interacts with other miRNAs or genes. For instances, the shoot regeneration inhibition is established by miR165/166 repression via AGO10 [27,29]; the leaf polarity is depended on the crosstalk between miR390-AGO7-*TAS3* and miR165/166-HD-ZIP III regulatory cascades [30]; the interaction of miR160 and miR165/166 is suggested to involve the dynamic balance between ABA and indole acetic acid (IAA) [31]. Additionally, the functions of miR165/166 have been revealed in other dicots, such as cotton, tomato and tobacco [32]. Compared with dicots, only miR166 family members are found in monocots. In rice, miR166 and its targets play important roles in stem xylem development, leaf rolling and drought resistance [32,33]. In addition, over-expressed miR166 or silenced *OsHB4* promotes cadmium tolerance [11].

Maize is not only a classic model plant for genetics research, but also a crucial crop for human food, animal feed, and industrial material [34]. Uncovering the genetic basis of important agronomic traits will be facilitated to productivity enhancement and quality improvement. In maize, miR166 and its target *Rld1* are mainly involved in leaf polarity regulation. *Rolled leaf 1* (*Rld1*) (a homologous gene of *Arabidopsis* HD-ZIP III transcription factor) is a target gene mutant of maize miR166, showing several developmental defects such as reduced stature, delayed flowering, and curled leaf [35,36]. Moreover, other leaf polarity mutants are documented in maize, including *lbl1* (*leafbladeless1*) (*Arabidopsis SGS3* homolog), *rgd2* (*ragged seedling2*) (*Arabidopsis ago7* homolog), *mwp1* (*milkweed pod1*) (*Arabidopsis KANADI* homolog) [36,37,38]. Intriguingly, miR166-*rld1* and miR390-*lbl1* are involved in the synthesis, transport, and action of miRNA and ta-siRNA, occupying in two contrasting regions, establishing an opposing gradient and maintaining the developmental pattern of the leaves [39,40,41]. Although the molecular mechanism of maize miR166 in leaf polarity has been resolved, its regulatory roles in abiotic stresses, flowering time, and kernel development are still less addressed.

Using short tandem target mimics (STTM) technology, our laboratory has developed a resource for miRNAs inactivation vectors and transgenic lines in both model and crop plants [32]. In the present study, STTM166 vector was selected for the resource to transform in the C01 background and characterize their developmental and environmental phenotypes. The transgenic plants exhibited rolling leaf, late flowering, small kernel size, defective vascular architecture, and enhanced several abiotic stresses tolerance. Transcriptomic analysis identified a number of genes that are expressed differently between STTM166 plants and the control. Our study provided the preliminary evidence of miR166 functions in plant development and abiotic stresses resistance in maize.

## 2. Results

### 2.1. Functional Blockage of Maize miR166 Family Using STTM Technology

**Figure 1:**
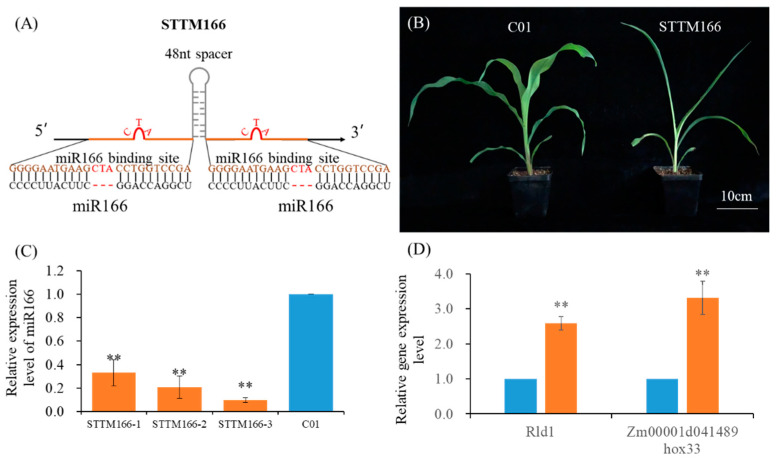
miR166 knockdown mediates its target genes up-regulated expression and leaf rolling in maize. (**A**) The diagram of STTM166 structure with a 48 nt length spacer and two non-cleavable miRNA binding sites. (**B**) Morphology of C01 and STTM166 seedlings. Bar = 10 cm. (**C**) qRT-PCR analysis of miR166 expression levels in C01 and STTM166 plants. (**D**) Analysis of miR166 target genes’ expression levels. U6 small nuclear RNA and *Actin* were used as the internal control of miR166 and target genes in qRT-PCR analysis, respectively. ** represents that the corresponding miR166 and target gene expression levels of STTM166 plants are very significantly different from the wild type *p* < 0.01, respectively. Bars show standard error of phenotypic values.

Increasing studies have demonstrated that STTM technology is an ideal approach for plant miRNA functional investigation [32,42]. A STTM resource of the model and crop plants was developed previously, in which maize pTF101.1-STTM166 vector was constructed [32]. The vector was designed as a 48 nt length spacer flanked by two uncleavable miR166 binding sites, which was driven by the 2 × 35S promoter (Figure 1A). Here, the pTF101.1-STTM166 vector was selected and transformed in maize inbred line C01 by Life Science and Technology Center, China Seed Group Co., Ltd. (Wuhan, China). The screened STTM166 plants exhibited classic severe leaf curling (Figure 1B). To further verify the transgenic plants, the expression levels of miR166 and its potential target genes were examined by qRT-PCR analysis (Figure 1C,D). In STTM166 plants, the expression levels of miR166 decreased significantly, only 9.7–33% comparing with the C01 plants. As expected, in the STTM166 plants, the potential miR166 target genes, *Rld1* and *Hox33*, were up-regulated for 1.5 and 2.3 folds. These results indicated that the miR166 family was successfully silenced by STTM, and consequently released their target genes.

### 2.2. miR166 Knockdown Mediates Maize Agronomic Traits Phenotypic Alterations

**Figure 2:**
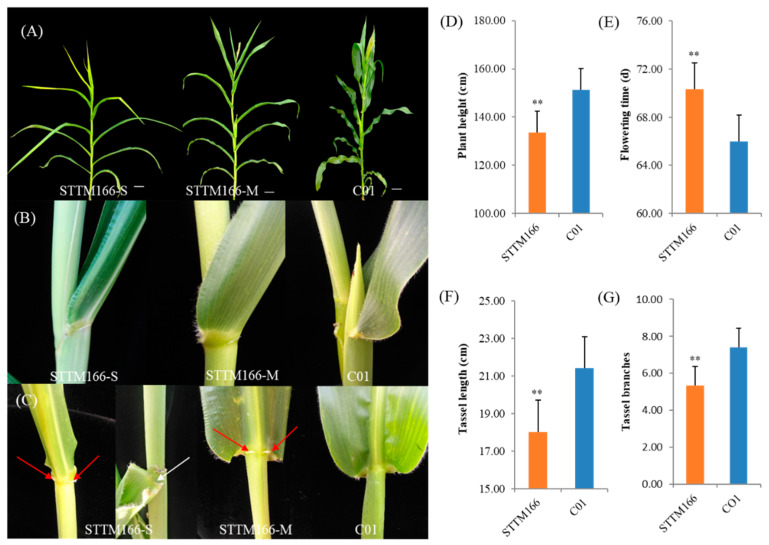
The phenotypic alterations of plant architecture, leaf shape, flowering and tassel branching mediated by miR166 inactivation. (**A**–**C**) The representative phenotypes of C01 and STTM166 of whole plants, leaf curling and leaf base. Bar represents 10 cm. STTM166-S, STTM166 plants with strong phenotypic alterations; STTM166-M, STTM166 plants with moderate phenotypic alterations. The leaf of STTM166 plants has an abaxial ligule (Red arrowhead); The STTM166-S plant developed a brittle leaf blade base and may resulted in abnormal leaf abscission (White arrowhead). (**D**–**G**) Comparisons of plant height, flowering time, tassel length and branches between C01 and STTM166 plants. In all case, ** represents that the corresponding phenotypic values of STTM166 plants are very significantly different from the wild type *p* < 0.01. Bars show standard error of phenotypic values.

**Figure 3:**
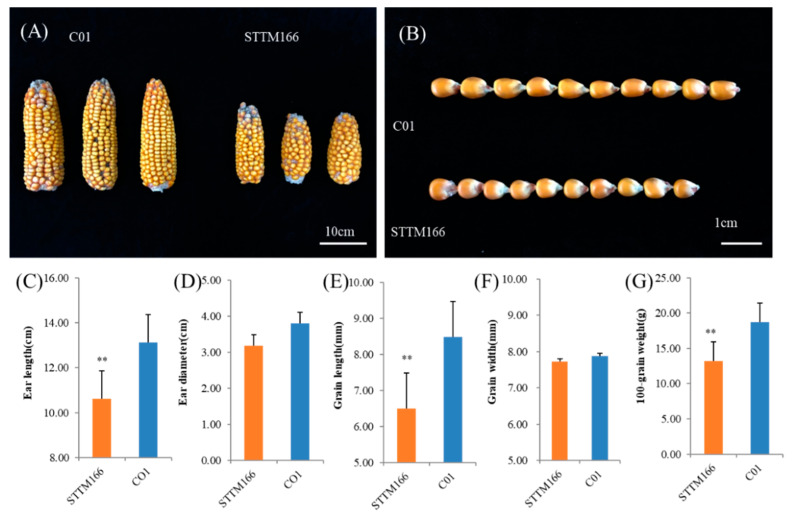
The phenotypic analysis of yield-related of maize STTM166 plants. (**A**) Comparison of ear-related traits between C01 and STTM166. Bar = 10 cm. (**B**) Comparison of grain size between C01 and STTM166. Bar = 1 cm. C-G. Comparison of yield-related traits between C01 and STTM166, including ear length (**C**), ear width (**D**), grain length (**E**), grain width (**F**), and 100 grain weight (**G**). In all case, ** represents that the corresponding phenotypic values of STTM166 plants are very significantly different from the wild type *p* < 0.01. Bars show standard error of phenotypic values.

Among the screened STTM166 plants, phenotypes of leaf curling and developmental phase change exhibited significant variance (Figure 2A–C). These STTM166 plants with strong leaf curling (STTM166-S) always display severe developmental phase transition delaying. Those STTM166 plants with moderate leaf curling (STTM166-M) exhibited weak alteration in developmental phase transition. It is implied that these two developmental processes are likely controlled by the same genetic pathway. Similar to the *Rld1* plants, the moderate and strong phenotypic STTM166 plants grew an abaxial ligule [35]. Strikingly, the STTM166-S plants developed a brittle leaf blade base, which may result in abnormal leaf abscission (Figure 2C). Moreover, STTM166 plants showed shorter plant height, later flowering time, and smaller tassel size in comparison with the C01 plants (Figure 2D–G). The reduced plant height and postponed flowering time are probably coincident with developmental transition delaying. The small tassel size of STTM166 plants hinted that miR166 is possibly involved in tassel development. Upon the yield-related traits, STTM166 plants showed small ears and grains (Figure 3). Compared with C01, the ears and grains of STTM166 were relatively shorter, and the 100-grain weight was relatively lighter. Furthermore, the ears of STTM166 plants displayed ambiguous ear rows. However, there were no significant differences in the ear diameter and grain width between STTM166 and C01. These results revealed that miR166 confers maize ear and grain development in a novel manner.

**Figure 4:**
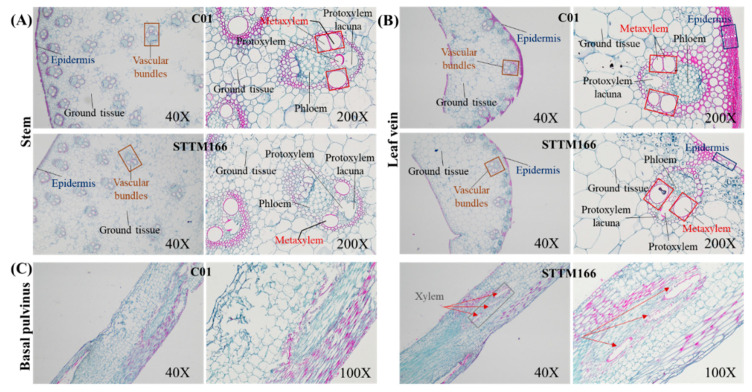
Cross sections of stem, leaf vein and basal pulvinus of C01 and STTM166 plants. (**A**) Cross sections of plant stem of C01 and STTM166 (40× and 200×). Vascular bundles are marked in orange frame and font; Epidermis is marked in dark-bule font; Metaxylem vessels are marked in red frame and font. (**B**) Cross section of plant leaf vein of C01 and STTM166 (40× and 200×). Epidermis is marked in dark-bule font; Metaxylem vessels are marked in red frame and font. (**C**) Cross section of basal pulvinus of C01 and STTM166 (40× and 200×). The xylem-like tissue is marked in gray frame and red arrows.

In rice, inactivation of miR166 altered vascular development in the stem [33]. To evaluate the effects of miR166 knockdown on maize vascular development, we performed histological analysis on the cross sections of stem and leaf vein of the C01 and STTM166 plants (Figure 4A,B). Compared with C01, the epidermis of STTM166 stems and leaf veins comprised of single/few layers of cells. The size of vascular bundles in STTM166 stems and leaf veins were extensively reduced. The diameter and number of metaxylem vessels in STTM166 stems and leaf veins were also significantly decreased. These results suggested that vascular development is largely coupled with between leaf veins and stems, and regulated by miR166. To explore the cause of brittle leaf blade base, we examined the microstructure of basal pulvinus in C01 and STTM166 plants (Figure 4C). The sectioning showed that STTM166 had abnormal vascular bundle-like tissues, but the wild type did not. Such a structure alteration in the basal pulvinus of STTM166 may allow the leaf to come off easily.

### 2.3. Maize STTM166 Displays Enhanced Abiotic Stresses Resistance

**Figure 5:**
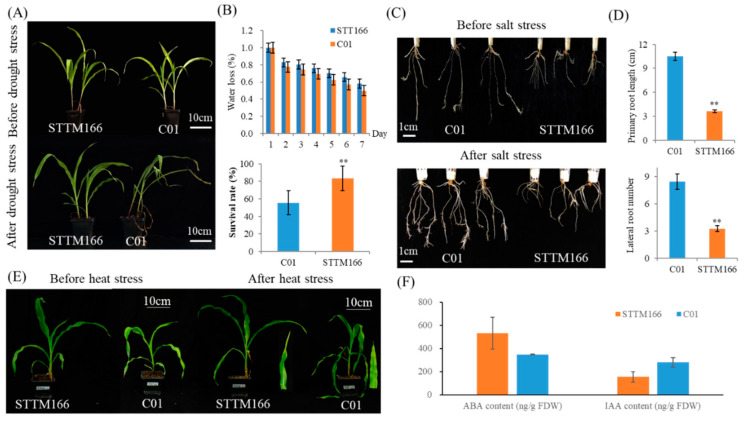
The responses of STTM166 plants to drought, salt, and heat stresses, as well as the contents of ABA and IAA. (**A**) Phenotypic alterations of C01 and STTM166 plants after one-week drought treatment. Bar = 10 cm. (**B**) Comparison of the water loss and survival rate of C01 and STTM166. ** represents that the corresponding phenotypic values of STTM166 plants are significantly different from the wild type *p* < 0.01. Bars show standard error of phenotypic values. (**C**) Phenotypic effects of salt stress on C01 and STTM166 root architecture. Bar = 1 cm. (**D**) Comparison of the root number of C01 and STTM166 plant after one-week salt stress treatment. ** represents that the corresponding phenotypic values of STTM166 plants are significantly different from the wild type *p* < 0.01. Bars show standard error of phenotypic values. (**E**) The phenotypic changes of C01 and STTM166 plants after four days heat stress. (**F**) Analysis of ABA and IAA contents of STTM166 and C01 plants. Bars show standard error of phenotypic values.

In *Arabidopsis* and rice, inactivation of miR165/166 enhances abiotic stresses tolerance, especially for drought tolerance [25,33]. To examine the response to main abiotic stresses, drought, salt and heat stress treatments were conducted on the maize STTM166-M seedlings (Figure 5). Among these seedlings, the expression levels of miR166 were remarkable reduced. When water was withheld for one week, C01 plants displayed severe arrested or even trapped in growth (Figure 5A). In contrast, STTM166 plants exhibited as much healthier and less impacted by the drought stress (Figure 5A,B). During water withholding, STTM166 plants showed lower water loss rate compared with that of C01 plants. When C01 and STTM166 plants were re-watered, less than 60% of C01 plants survived and continued to grow.

Two-week-old maize STTM166-M plants displayed shorter and less roots as compared with the wild-type, indicating that miR166 involves root architecture maintenance (Figure 5C,D). When these seedlings were subjected to one-week salt stress treatment by 200 mM NaCl solution, the root system of STTM166 plants showed almost no effect (Figure 5C,D). In contrast, C01 plants showed many lateral roots grown, which implied that C01 seedlings were more sensitive to salt stress. In addition, three-week-old maize seedlings were subjected to high temperature treatment (14 h of light at 38 °C and 10 h of darkness at 28 °C) for 4 days (Figure 4E). The leaves of C01 plants exhibited remarkable water loss, but no obvious effects on the STTM166 plants. These results revealed that miR166 inactivation enhances abiotic stress tolerance in maize early development.

In plants, ABA and auxin are two key regulators in response to abiotic stress [43,44]. *Arabidopsis* miR165/166 plays critical roles in ABA homeostasis, which is probably associated with the abiotic stress resistance of STTM165/166 plants [25]. In the present study, the ABA and IAA contents of C01 and STTM166 plants were measured to explore the effects of miR166 knockdown in maize. Compared with the C01 plants, STTM166 plants exhibited increased ABA content, whereas decreased IAA content (Figure 5F).

### 2.4. Differentially Expressed Genes (DEGs) in C01 and STTM166 Plants Revealed by RNA-Seq

**Figure 6:**
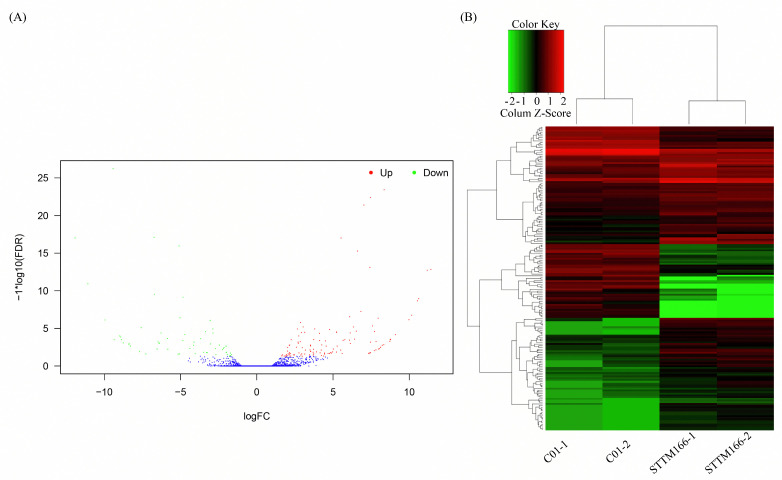
The differentially expressed genes (DEGs) between C01 and STTM166 plants. (**A**) Gene expression classification of DEGs. Red dots indicate up-regulated genes and green dots for down-regulated genes (Significant level: Padj <0.05, Log2FoldChange >1). (**B**) Heat map of the DEGs between C01 and STTM166 plants.

To identify the miR166 regulated genes, leaf samples of C01 and STTM166-M plants (each for two biological replicates) were collected and constructed libraries for RNA sequencing. Each replicate of STTM166 consisted of the ear leaf samples from three plants that belong to the same transformation event. All the samples displayed significantly miR166 down-regulated. By transcriptomic analysis, a total of 178 DEGs were discovered between C01 and STTM166 plants, including 118 up-regulated genes and 60 down-regulated genes (Figure 6).

**Figure 7:**
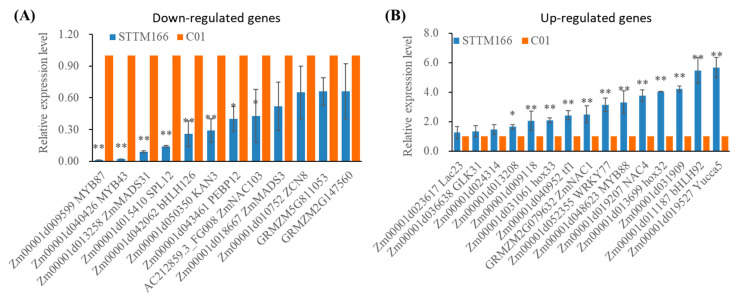
The expression analysis of DEGs between C01 and STTM166 by qRT-PCR. (**A**) The expression of down-regulated genes; (**B**) The expression of up-regulated genes. *Actin* was used as an internal control. * and ** represents that the corresponding gene expression levels of STTM166 plants are significantly different from the wild type *p* < 0.05 and *p* < 0.01, respectively. Bars show standard error of phenotypic values.

Among the DEGs (Table 1; Figure 7), 6 homeobox-leucine zipper protein encoding genes, including two miR166 target genes, were significantly up-regulated in STTM166 plants. Two key leaf polarity establishment regulators encoding genes, *KAN1* and *KAN3*, showed remarkably down-regulated that probably contributed to the curling leaf in STTM166. There were also several flowering related genes displayed significantly differences between C01 and STTM166 plants, such as the up-regulated genes *Zm00001d036242* (Flowering locus T protein), *Zm00001d027957* (MADS68); the down-regulated genes *Zm00001d018667* (MADS3), *ZCN8*, *Zm00001d043461* (Flowering locus T protein). This implied that miR166 regulates maize flowering time in a complicated manner. In the up-regulated genes of STTM166 plants, some of them are likely involved in abiotic stress response, such as *Zm00001d021777* (Vacuolar protein 8-like), *ZmWRKY14*, *ZmWRKY77*, *ZmNAC7*, *ZmNAC1*, *ZmNAC4*, and *GRMZM2G088964* (Probable potassium transporter 17) (Table 1; Figure 7B). These genes are probably associated with the enhanced abiotic stress tolerance in STTM166 plants. Compared with the gene expression in C01 plants, a cellulose synthesis related gene (*Zm00001d036900*) displayed up-regulated expression, but another gene (*Cellulose synthase-like protein e6*) showed down-regulated expression, the two cellulose synthesis related genes are possible to regulate vascular bundles patterning. Strikingly, the encoding gene of Argonaute 12-like protein displayed up-regulated expression in STTM166, which may be involved in small RNA mediated gene silencing. Additionally, several hormone related genes showed significant differences between C01 and STTM166, such as *Zm00001d037343* (ABA-induced protein), *Zm00001d042809* (Auxin transporter-like protein 1), *Zm00001d004248* (Cytokinin-o-glucosyltransferase 2), and *Yucca5* (Indole-3-pyruvate monooxygenase). The expression alterations of these genes were mostly consistent with the changes of ABA and IAA content in STTM166 plants.

**Figure 8:**
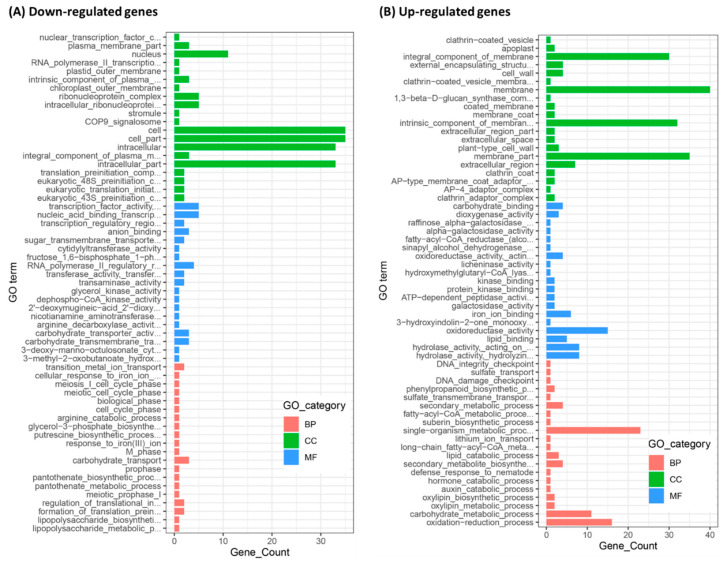
Gene ontology analysis of the screened DEGs between C01 and STTM166 plants. (**A**) Gene ontology analysis of the down-regulated genes; (**B**) Gene ontology analysis of the up-regulated genes. BP, biological process; CC, cell component; MF, molecular function.

**Figure 9:**
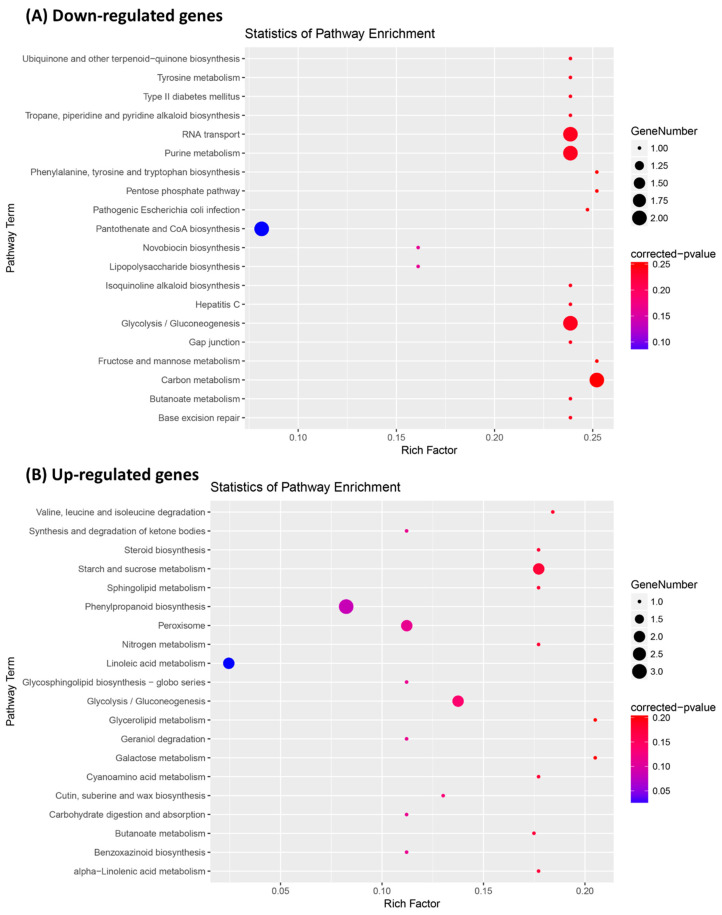
Kyoto encyclopedia of genes and genomes (KEGG) enrichment analysis of DEGs between STTM166 and C01. (**A**) The KEGG enrichment of the down-regulated genes; (**B**) The KEGG enrichment of the up-regulated genes.

The screened DEGs between C01 and STTM166 were subjected to gene ontology (GO) analysis, most of these genes were identified to be associated with cellular component organization or biogenesis, few genes were enriched in biological process and molecular function (Figure 8). The down-regulated genes were mainly associated with cell, nucleus and intercellular component biosynthesis and arrangement pathways. In turn, most of those up-regulated genes were mainly associated with cell membrane component, oxidoreductase activity, hydrolase activity, single organism metabolic process, carbohydrate metabolic process, and oxidation reduction processes. Kyoto encyclopedia of genes and genomes (KEGG) analysis indicated that those down-regulated genes were strongly enriched in and associated with RNA transport, purine metabolism, glycolysis or gluconeogenesis, and carbon metabolism (Figure 9). In contrast, those up-regulated genes were largely enriched in and associated with starch and sucrose metabolism, phenylpropanoid biosynthesis, peroxisome, and glycolysis or gluconeogenesis. These results revealed that miR166 primarily regulates cell component biogenesis and organization, stress resistance, and carbohydrate metabolism, thereby defining the leaf polarity defect, abiotic stress tolerance, and inferior yield-related traits.

### 2.5. Expressions of Key Regulatory Genes in ABA and Auxin Biogenesis and Signaling Pathways

**Figure 10:**
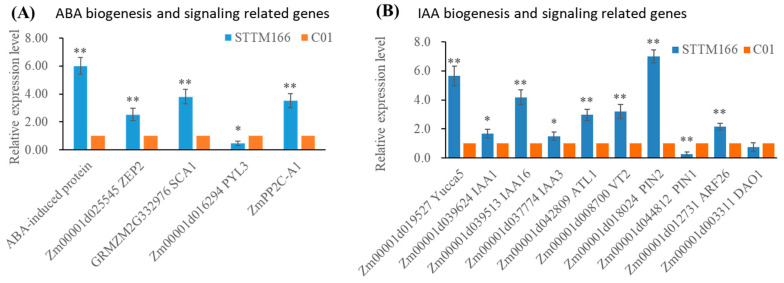
The expression levels of the key genes involved in ABA and IAA biogenesis and signaling pathways. (**A**) The expression of ABA biogenesis and signaling related genes; (**B**) The expression of IAA biogenesis and signaling related genes. *Actin* was used as an internal control. * and ** represents that the corresponding gene expression levels of STTM166 plants are significantly different from the wild type *p* < 0.05 and *p* < 0.01, respectively. Bars show standard error of phenotypic values.

In STTM166 plants, miR166 inactivation led to ABA content induction and IAA content reduction. To identify the expression pattern of some involving genes, ABA and auxin biogenesis and signaling pathways genes were further tested (Figure 10). Among the examined ABA biogenesis and signaling genes in STTM166 plants, only the ABA receptor encoding gene, *Zm00001d016294* (*PYL3*), exhibited down-regulated expression, while those ABA biogenesis, signaling, and induced protein encoding genes were greatly up-regulated. Notably, most auxin biogenesis, signaling, and responsive protein encoding genes were greatly increased, only an auxin transporter encoding gene showed decreased expression level. These results are consistent with those corresponding gene expression alterations in *Arabidopsis* STTM165/166 plants, which further implied miR166 mediated ABA and auxin homeostasis to involve complicated regulatory mechanisms.

## 3. Discussion

Extensive studies have demonstrated the crucial roles of miRNAs in plant development and stress response. For instance, functions of miR165/166 in various plant processes have been widely explored in dicots and monocots [5,15,21,24,25,26,32,33,35,36,45,46]. Like other plants, the role of maize miR166 in leaf polarity determination has been early investigated [35,41]. However, other functions of maize miR166 were still largely unknown. Using STTM technology, we have developed a resource for major miRNA inactivation in model and crop plants, including a collection of STTM plasmids and transgenes [32]. In our previous study, STTM was applied to knock down maize miR166 and cause defective leaf polarity. To further explore the functions of maize miR166, we generated and charactered new STTM166 transgenic lines. In this study, the STTM166 plants not only displayed defective leaf polarity, but also exhibited overall phenotypic alterations, such as developmental phase transition, flowering time, plant height, tassel size, ear length, grain size, vascular patterning, leaf base tissue specifying, root architecture, and abiotic stress tolerance. More importantly, miR166 inactivation mediated ABA content increase and IAA content decrease.

Plant hormones, such as ABA and auxin, regulate many aspects of plant development and stress response. In *Arabidopsis*, miR165/166 was suggested to confer multiple abiotic stress resistance through an ABA-dependent pathway [25,31]. Of which, miR165/166 triggers the ABA homeostasis through the regulation of its targets and *BG1* expression levels. In rice, knockdown of miR166 improved plant drought resistance by causing rolled leaf and altered xylem [33]. Compared with the wild-type plants, ABA levels in rice STTM166 displayed no significant difference. These results revealed that the regulatory mechanism in miR165/16-mediated abiotic stress resistance may differ between dicots and monocots. In the present study, maize miR166 inactivation mediated ABA levels elevation and drought resistance. However, the underlying regulatory mechanism still needs to be explored. More strikingly, miR165/166 has been proved to participate in IAA level regulation via the interplay of *HD-ZIP III* and *KANADI* genes in *Arabidopsis* and rice [40]. In *Arabidopsis*, the absence of miR165/166 resulted in elevated IAA level [31]. However, here, the IAA level displayed down-regulated in maize STTM166 plants, most auxin biogenesis and signaling related genes were up-regulated. Such difference between *Arabidopsis* and maize might be caused by ABA and IAA interaction. Further, the ABA and IAA interaction can be a putative pathway to reshape the root architecture in STTM166 plants [47].

Abiotic stress largely limits plant growth and development, even lead to death. The primary signal of drought, salt, and temperature stresses are osmotic stress and ionic or ion-toxicity effects [48]. The hormone ABA is an important regulator in abiotic stress response [49]. The STTM165/166 plants of *Arabidopsis* and rice were both displayed superior drought resistance [25,33]. In the present study, three superior characters of STTM166 plants probably contribute the abiotic stress resistance. Firstly, miR166 knockdown resulted in elevated ABA level. Secondly, the decreased vascular diameter and number, reduced primary and lateral roots, as well as the rolled leaves can lower water loss. Thirdly, those up-regulated genes were strongly enriched in cell membrane system components biosynthesis, which are possible to benefit for enhancing stress tolerance.

In plants, vascular tissues are important for water and nutrition transporting, as well as physical support of stem upright [50]. Considerable studies revealed that vascular development is tightly controlled by hormonal response, peptide signaling, and transcriptional regulation [51,52]. In *Arabidopsis* and rice, miR165/166 has been reported to confer vascular development through binding its target genes [33,53,54]. In the present study, the diameter and number of metaxylem vessels was significantly decreased in stems and leaf veins of STTM166 plants. Such alterations were consistent with the down-regulated expression of cell and intercellular components related genes that greatly enriched in GO analysis. Two cellulose synthesis related genes displayed opposite expression pattern, which are likely to contribute to the xylem biogenesis and patterning, as well as the epidermis development. Previous studies revealed that IAA serves as an important players in the initiation of vascular procambial cells [50]. The IAA levels altered by miR166 inactivation provides an alternative pathway for vascular development. The abiotic stress resistance and brittle leaf basal showed in STTM166 plants were possibly partly determined by vascular structure alterations.

As maize plants transit from the vegetative stage to reproductive stage, the shoot apical meristem converts to the male inflorescence meristem, and the axillary meristem converts to the female inflorescence meristem [55]. The two kinds of inflorescence meristem (IM) elongate and produce the spikelet-pair meristem (SPM), each SPM then builds two spikelet meristems (SMs), SM further give rise to floral meristem (FM), and finally develop to tassel and ear. So far, several genes have been identified to control maize tassel and ear development, such as auxin transporter gene *PINOID* [56], *CLAVATA-WUSCHEL (CLV-WUS)* [57,58], SBP-box transcription factor genes *tasselsheath4* (*tsh4*) [59], *unbranched2* (*ub2*), and *ub3* [60], APETALA2 (AP2) transcription factor genes *indeterminate spikelet1* (*ids1*) and *sister of indeterminate spikelet1* (*sid1*) [61]. In addition, miR165/166 has been found to modulate apical meristem formation through binding *HD-ZIP III* genes or recruited by Argonaute10 (AGO10) in *Arabidopsis* [27,53,62]. In maize, miR166 was uncovered to regulate spikelet meristems development on the tassel central spike by a Argonaute18b (AGO18b) dependent gene silencing pathway [63]. In the present study, STTM166 plants displayed shorter tassels with fewer branches, smaller ears with confusing ear rows, and decreased grain size. It can be speculated that miR166 confers maize tassel and ear development through auxin-dependent pathway, AGO18b-dependent gene silencing, or interacting with miR156 or miR172. Strikingly, an Argonaute12-like protein (AGO12, AGO1/AGO5/AGO10 subgroup) gene was up-regulated in STTM166, which is implied miR166 to interact with AGO12 that similar to the interaction of miR165/166 and AGO10 in *Arabidopsis*. Additionally, the miR166 mediates tassel development regulation maybe correlates with the flowering time determination.

Drought, salt and heat stresses largely restrict maize production in some areas of the world. In the present work, we generated a series of miR166 knockdown lines that exhibited distinct degrees of phenotypic alteration. These STTM166 plants usually have enhanced abiotic stress resistance, which can be applied for breeding elite inbred lines and varieties with superior stress resistance. However, those defective phenotypes in STTM166-S lines, such as rolled leaves and inferior yield-related traits, might bring some negative effects. Thus, those STTM166-S lines are not suitable for maize breeding. Only those STTM166 lines with moderate phenotypes facilitate to meet the requirements of maize breeders. Those STTM166-M lines usually have relative enhanced stress resistance, but weak defective phenotypes that slightly affects the yield. In conclusion, our study defined the functions of maize miR166, and shed insights on applying STTM technology in agronomic traits improvement. However, there were several issues still need to be addressed before applied in maize production. Firstly, the favorable level of miR166 still need to be identified in future, which largely determines the application of miR166 in agronomic traits improvement. Secondly, the regulatory networks of miR166 in maize development and stress response have not been fully resolved. Thirdly, miR166 is probably in the interaction with other miRNAs, which is largely unknown.

## 4. Materials and Methods

### 4.1. Maize STTM166 Construction

In our previous study [32], maize STTM166 binary expression vector was constructed for miR166 inactivation referring to the method of STTM vector construction by Tang et al. [28]. First, STTM166 was cloned into the intermediate vector pOT2-poly-cis, and the recombinant vector pOT2-STTM166 was identified. Then, the binary expression vector pTF101.1 was selected for maize STTM transformation, whom was modified to add a PacI cleavage site. Finally, the STTM166 element on the recombinant vector pOT2-STTM166 was cloned into the modified binary expression vector pTF101.1/PacI, resulting in pTF101.1-STTM166. The final STTM166 construct was screened by Spectinomycin resistance, and confirmed by DNA sequencing using the STTM common real primer.

Common real primer:

STTM common real-PF: 5′-CATTTGGAGAGGACAGCCCAAG-3′
STTM common real-PR: 5′-CTGGTGATTTCAGCGTACCGAA-3′

### 4.2. Plant Transformation, Transgenic Plants Screening, Genotyping and Phenotyping

The constructed pTF101.1-STTM166 binary expression vector was transformed in maize (Life Science and Technology Center of China Seed Group Co., Wuhan, China). The obtained maize STTM166 transgenic were screened by Basta resistance, and genotyping using the STTM common real primer. Then, the expression level of miR166 and its target genes were tested by qRT-PCR analysis (All the primers were list in Table S1). Maize STTM166 plants were self-pollinated for three times for reproduction and getting the homozygous transgenic lines. The phenotypic alterations of STTM166 plants were investigated in field, including plant architecture, flowering time, leaf rolling, tassel architecture, and yield-related traits. Phenotypic investigations were conducted in three environments. At flowering time, the ear leaf samples of STTM166-M and the transgenic background (C01) plants were randomly collected for transcriptome sequencing and qRT-PCR analyses. These field collected leaf samples were immediately placed in liquid nitrogen and then stored in −80 °C condition.

### 4.3. Histological Analysis

For histological analysis, at flowering time, ear leaves and the third stem internodes above ground of C01 and STTM166-M plants were collected with three biological replicates from different transformation events. Selected and cleaved the middle part of these collected maize leaf and stem samples into small pieces, further placed in precooled 70% FAA solution. After the tube air pumped, the samples were fixed overnight at 4 °C in FAA solution, and further to dehydrate in graded ethanol series approach. The samples were then re-dehydrated and transparent treated, and embedded in paraffin wax (Sigma-Aldrich). Further, the samples were cleaved into 4-μm sections using a Leica RM 2265 programmable rotary microtome (Leica Microsystems). After being stained with 0.05% toluidine blue, the sections were photographed using an Olympus IX73 microscope (Olympus, Tokyo, Japan).

### 4.4. Total RNA Extraction, Transcriptome Sequencing Analysis, qRT-PCR

For transcriptomic sequencing, leaf samples of wild-type (C01) and STTM166 with three replicates, each replicate includes three randomly selected plants that belong to the same transformation event, were collected at flowering time. Total RNA of all the samples were extracted using Trizol reagent (Invitrogen, MA, USA) according to the manufacturer’s instructions. Next, the RNA quality and quantity were tested by Nanodrop 2000 (Thermo Scientific, MA, USA), agar gel electrophoresis, and Agilent 2100 Bioanalyzer System (Agilent, CA, USA). Of the tested RNA samples, two replicates for each plant material were selected for cDNA library preparation.

Using the TruSeq RNA Sample Prep Kit (Illumina, CA, USA), four cDNA libraries (two replicates for wild-type C01 and STTM166) were prepared as the following steps. Firstly, the total RNA samples were digested by DNase I and purified by oligo (dT) beads for obtaining high quality mRNA. Then, the obtained mRNAs were fragmented and reverse transcribed into cDNA, followed by 3′end separation, poly (A) tail addition and adapter ligation. Finally, the synthesized cDNA was used as templates for PCR amplification and constituting cDNA libraries. Next, the libraries were sequenced by the Illumina Hiseq2500 platform (Berry Genomics, Beijing, China). The original transcriptomic sequencing data in fastq format has been uploaded to NCBI SRA database (Accession number: PRJNA675730, https://www.ncbi.nlm.nih.gov/sra/PRJNA675730).

The obtained transcriptomic sequencing data was analyzed as the following procedure. Firstly, Trimmomatic software V0.36 (FSU, FL, USA) [64] was used to analyzed raw reads of the sequencing data to remove adapters and low-quality bases. Secondly, the acquired clean reads were aligned to the maize reference genome (B73 RefGen_V4.42, http://ensembl.gramene.org/Zea_mays/Info/Index) using the HISAT2 V2.1.0 (Default parameters setting) [65]. The transcript abundance was further calculated as fragments per kilobase of exon per million fragments mapped (FPKM). Then, DEGs were identified using DESeq2 software V1.22.2 (http://www.bioconductor.org/packages/release/bioc/html/DESeq2.html) [66], with the threshold settings of |log2 fold change| ≥ 1 and *p*-values < 0.05. Finally, the gene ontology (GO) and Kyoto Encyclopedia of Genes and Genomes (KEGG) enrichment analyses were conducted using the maize profile database (org.Zeamays.eg.sqlite) in the clusterProfiler software V3.10.1 (http://bioconductor.org/packages/release/bioc/html/clusterProfiler.html) [67], and Annotation Hub (V2.14.5) R package (https://bioconductor.org/packages/release/bioc/html/AnnotationHub.html) [68]. The enrichment analyses use the ensemble database to convert the gene number (maizegbdId) to the corresponding Entrez ID.

To evaluate the expression levels of miR166 and its regulated genes, as well as to verify the transcriptome sequencing results, qRT-PCR analysis was performed. The relative expression level of miR166 was detected using the Mir-X™ miRNA qRT-PCR SYBR^®^ Kit (Takara, Dalian, China). U6 small nuclear RNA was used as the internal reference for miR166 qRT-PCR. In regard to miR166 target genes and regulated genes, total RNA of the tested samples was reverse transcribed using the PrimeScript™ RT reagent kit with gDNA Eraser (Perfect Real Time) (Takara, Dalian, China). Then, the cDNA of the genes to be detected were quantified using the CFX96 Touch™ Real-Time PCR Detection System (Bio-Rad, CA, USA) and the SYBR^®^ Premix EX Taq™ II (Tli RNaseH Plus) Kit (Takara, Dalian, China). *Actin* was used as internal references for key genes qRT-PCR, respectively. The relative expression levels of miR166 and the tested genes were calculated based on the 2-ΔΔCt method [69]. All the tested genes and miR166 were quantified by at least three biological replicates. The qRT–PCR primers are listed (Supplementary Table S1).

### 4.5. Plant Hormone Content Measurement

Those frozen dried leaf samples of C01 and STTM166-M plants for qRT-PCR analysis were also subjected to measure plant hormone contents [70]. Using high-performance liquid chromatography (HPLC) method, the IAA and ABA contents were tested for three biological replicates and four technical replicates (Suzhou Comin Biotechnology Co. Ltd., Suzhou, China). The hormone contents were calculated per freeze dry mass.

### 4.6. Drought, Salt, Heat Stress Treatment

After seed germination, C01 and STTM166-M seeds were transferred into pots (10 cm × 10 cm) with 280 g soil. The plants were grown in plant chamber (RTQP-1000, TOP Instrument, Hangzhou, China) under 16 h of ~1200 µmol photons m^−2^ s^−1^ PPFD at 28 °C and 8 h of darkness at 25 °C, and ~70% relative humidity. The plants were well watered. To conduct drought and salt stress treated experiments, two-week-old pot-grown maize seedlings were subjected to water-limitation and NaCl solution (200 mM) treatments for three replicates [71,72]. In drought treatment, each replicate includes 12 seedlings of C01 and STTM166-M for 7 days water withholding. During the treating process, the water loss rate was measured for each day by weighting the plant pots. On the 8th day of water-limitation, the treated maize seedlings were re-watered, and the survival rate were recorded on the 8^th^ day after re-watering. In salt treatment, each replicate includes 12 seedlings of C01 and STTM166, grown in 1/2 MS medium solution with 200 mM NaCl for 7 days. After salt stress, primary and lateral roots of C01 and STTM166 plants were counted. For heat stress treatment, three-week-old pot-grown maize seedlings of C01 and STTM166 were selected to grow in a plant incubator (RTQP-1000, TOP Instrument, Hangzhou, China) for 4 days, the growth condition was set as 16 h of ~1200 µmol photons m^−2^ s^−1^ PPFD at 38 °C and 8 h of darkness at 28 °C, ~70% relative humidity. These treated plants were well-watered without other stress.

### 4.7. Statistical Analysis

All the collected data from phenotypic analysis, qRT-PCR analysis data and stress treatment experiments were subjected to one-way variance analysis (ANOVA) and Student’s t-test using software SPSS 22.0 (IBM, NY, USA). *p* < 0.05 indicates the statistical differences to reach the significant different level, *p* < 0.01 for very significant different levels.

## Figures and Tables

**Table 1:** The screened key DEGs between C01 and STTM166 plants in transcriptomic analysis.

Gene	logFC	*p*-Value	C01-FPKM	STTM166-FPKM	Description
Up-regulated genes					
*Zm00001d040952*	7.96	4.86 × 10^−10^	0.03	8.26	Homeobox-leucine zipper protein TF1
*Zm00001d013699*	5.52	3.07 × 10^−21^	1.91	103.01	Homeobox-leucine zipper protein HOX32
*Zm00001d036638*	4.9	1.32 × 10^−4^	0.1	2.79	MYB family transcription factor
*Zm00001d021777*	4.55	1.01 × 10^−5^	0.19	5.5	Vacuolar protein 8-like
*Zm00001d026510*	4.38	1.77 × 10^−3^	0.1	1.92	Transcription factor IBH1
*Zm00001d041489*	3.74	8.30 × 10^−7^	0.43	5.61	Homeobox-leucine zipper protein HOX33
*Zm00001d036242*	3.71	5.39 × 10^−3^	0.25	3.11	Flowering locus T protein
*Zm00001d031061*	3.46	2.66 × 10^−4^	0.19	2.03	Homeobox-leucine zipper protein HOX33
*Zm00001d033518*	3.22	5.02 × 10^−3^	0.06	0.54	Protein Argonaute 12-like
*GRMZM2G083717*	3.19	6.80 × 10^−3^	0.26	2.51	Probable WRKY transcription factor 14
*Zm00001d037343*	3.15	1.98 × 10^−4^	1.35	10.35	ABA-induced protein
*Zm00001d027957*	2.95	5.41 × 10^−3^	3.83	33.24	MADS-box transcription factor 47-like isoform x1
*Zm00001d033246*	2.87	2.13 × 10^−9^	5.16	36.31	Homeobox-leucine zipper protein HOX32
*AC212859.3_FG008, NAC103*	2.85	1.30 × 10^−3^	0.73	5.21	NAC domain-containing protein 7
*Zm00001d035211*	2.4	7.24 × 10^−4^	3.58	15.93	E3 ubiquitin-protein ligase RGLG2
*Zm00001d027317, rld2*	2.15	7.00 × 10^−6^	3.78	16.54	Homeobox-leucine zipper protein HOX10
*Zm00001d036900*	1.81	7.83 × 10^−3^	1.88	6. 41	Cellulose synthase a catalytic subunit 11
*Zm00001d048527, rld1*	1.59	2.34 × 10^−3^	2.96	8.84	Homeobox-leucine zipper protein HOX10
*GRMZM2G079632*	1.54	4.79 × 10^−3^	7.83	22.4	NAC1
*GRMZM2G088964*	1.52	7.89 × 10^−3^	3.84	11.48	Probable potassium transporter 17
*Zm00001d042809*	1.15	9.42 × 10^−3^	12.47	27.61	Auxin transporter-like protein 1
Down-regulated genes					
*Zm00001d036613*	−6.73	1.98 × 10^−21^	48.8	0.45	Receptor-like protein kinase AT3G47110
*Zm00001d050350, KAN3*	−5.16	2.11 × 10^−4^	3.71	0.09	Probable transcription factor Rl9
*Zm00001d023311*	−5.1	4.28 × 10^−20^	17.08	0.5	Disease resistance protein RGA3
*Zm00001d042062*	−4.72	1.49 × 10^−7^	20.16	0.8	Transcription factor bHLH100-like
*Zm00001d018667*	−3.42	5.64 × 10^−4^	7.95	0.71	Mads-box transcription factor 15
*Zm00001d035343*	−3.37	1.73 × 10^−3^	6.17	0.5	Wall-associated receptor kinase 2-like
*Zm00001d032249*, *KAN1*	−3.30	1.13 × 10^−4^	5.76	0.56	Probable transcription factor Rl9
*Zm00001d020495*	−2.87	6.20 × 10^−4^	14.87	2.03	Probable WRKY transcription factor 40
*Zm00001d011847*	−2.04	3.02 × 10^−3^	267.89	68.32	Transcription factor bHLH100-like
*Zm00001d010752, ZCN8*	−1.79	4.14 × 10^−4^	91.07	26.51	Flowering locus T like protein
*Zm00001d03099, bZIP111*	−1.58	5.45 × 10^−3^	746.06	237.9	bZIP transcription factor superfamily protein
*GRMZM2G014558*	−1.42	9.32 × 10^−3^	123.94	47.05	Cellulose synthase-like protein E6
*Zm00001d037354*	−1.38	3.00 × 10^−3^	31.73	12.35	Calmodulin-binding heat-shock protein
*Zm00001d004248*	−1.29	9.04 × 10^−3^	99.98	41.04	Cytokinin-o-glucosyltransferase 2

## Supplementary Materials

The following are available online at https://www.mdpi.com/1422-0067/21/24/9506/s1, Table S1. Primers used for qRT-PCR analysis in this study.

## Abbreviations

STTM	Short Tandem Target Mimic
miR166	microRNA166
HD-ZIP III	Class III homeodomain/Leu zipper
DEGs	Differentially expressed genes
